# Parkin-mediated ubiquitination inhibits BAK apoptotic activity by blocking its canonical hydrophobic groove

**DOI:** 10.1038/s42003-023-05650-z

**Published:** 2023-12-12

**Authors:** Peng Cheng, Yuzhu Hou, Mingxing Bian, Xueru Fang, Yan Liu, Yuanfang Rao, Shuo Cao, Yanjun Liu, Shuai Zhang, Yanke Chen, Xu Dong, Zhu Liu

**Affiliations:** 1 National Key Laboratory of Crop Genetic Improvement, Hubei Hongshan Laboratory, Huazhong Agricultural University, Wuhan, 430070 China; 2 College of Biomedicine and Health, Huazhong Agricultural University, Wuhan, 430070 China; 3 Wuhan Institute of Physics and Mathematics, Innovation Academy for Precision Measurement Science and Technology, Chinese Academy of Sciences, Wuhan, 430071 China; 4 Present Address: State Key Laboratory of Biocatalysis and Enzyme Engineering, College of Life Sciences, Hubei University, Wuhan, 430074 China

**Keywords:** Biochemistry, Biophysics

## Abstract

BAK permeabilizes the mitochondrial outer membrane, causing apoptosis. This apoptotic activity of BAK is stimulated by binding prodeath activators within its canonical hydrophobic groove. Parkin, an E3 ubiquitin (Ub) ligase, can ubiquitinate BAK, which inhibits BAK apoptotic activity. However, the molecular mechanism underlying the inhibition of ubiquitination remains structurally uncharacterized. Here, we utilize truncated and soluble BAK to construct a mimetic of K113-ubiquitinated BAK (disulfide-linked Ub^G76C^ ~ BAK^K113C^) and further present its NMR-derived structure model. The classical L8-I44-H68-V70 hydrophobic patch of the conjugated Ub subunit binds within the canonical hydrophobic groove of BAK. This Ub occludes the binding of prodeath BID activators in the groove and impairs BID-triggered BAK activation and membrane permeabilization. Reduced interaction between Ub and BAK subunits allows BID to activate K113-ubiquitinated BAK. These mechanistic insights suggest a nonsignaling function of Ub in that it directly antagonizes stimuli targeting Ub-modified proteins rather than by recruiting downstream partners for cellular messaging.

## Abstract (web summary)

An NMR study on K113-ubiquitinated BAK complex reveals that the conjugated ubiquitin subunit binds to the canonical hydrophobic groove of BAK, which prevents the binding of prodeath BH3 activators and impairs BH3-induced BAK apoptotic activity.

## Introduction

The pro-apoptotic BCL-2 proteins BAK and BAX are essential effectors of mitochondrial apoptosis. In response to apoptotic stimuli, the BAK and BAX monomers homo-oligomerize into proteolipid pores within the mitochondrial outer membrane (MOM)^1–4^. These pore-forming BAK and BAX oligomers permeabilize MOM, resulting in cytochrome *c* efflux and thus apoptosis^5–7^. BAK and BAX oligomerization is triggered by weak interactions with prodeath activators such as cleaved BID, BIM and PUMA derived from BH3-only proteins (BH3s)^8–10^. Mechanistic studies indicate that these BH3 activators can transiently bind to the canonical hydrophobic groove of BAK and BAX^11–17^. The transiently interactions with BH3 activators induce subsequent conformational changes that facilitate the oligomerization of BAK and BAX monomers^18–24^. Other BCL-2 members, the anti-apoptotic proteins BCL-2, BCL-X_L_ and MCL-1, are thought to inhibit the apoptotic functions of BAK and BAX by directly binding and preventing their oligomerization^25–27^ or by sequestering BH3 activators into inert complexes^28–32^. Moreover, a novel regulatory function limiting BAK activity has been proposed wherein Parkin can ubiquitinate BAK to impair its activation and oligomerization^33^.

Parkin is a crucial E3 ubiquitin (Ub) ligase that plays important roles in the clearance of damaged mitochondria. This E3 ligase builds Ub moieties on mitochondrial outer membrane proteins that recruit autophagy receptors, ultimately triggering mitophagy^34,35^. Loss-of-function mutations in Parkin are associated with early-onset Parkinson’s disease^36,37^. Jonathan Bernardini and co-workers found that following mitophagic stress, BAK was ubiquitinated by Parkin^33^. This modification inhibits BAK activity and suppresses apoptosis, thereby allowing the effective clearance of damaged mitochondria via mitophagy^33^. The Parkin-mediated ubiquitination of BAK therefore provides a new regulatory modality to fine-tune apoptosis^38–40^. BAK contains two lysine residues, one positioned adjacent to its hydrophobic groove at position 113 and the other located in the C-terminus at position 210. As K210 is localized to the mitochondrial inter-membrane space and likely inaccessible to Parkin^41^, the authors speculated that K113 is the primary ubiquitination site^33^, and further validated this hypothesis by performing a cell-based assay of the K210R mutation^33^. These existing functional studies indicated that BAK K113 ubiquitination might obscure its hydrophobic surface groove and therefore, potentially impair binding with BH3 activators;^33^ however, direct evidence is lacking. The absence of the K113-ubiquitinated BAK complex structure limits our mechanistic understanding of how ubiquitination inhibits BAK activation.

The regulation of BAK apoptotic activity is fundamental in programmed cell death. While the mechanisms of BAK activation/inhibition by BCL-2 family members have been extensively studied, the newly identified regulation of BAK apoptotic function by ubiquitination inhibition remains mechanistically uncharacterized. In this work, we constructed the truncated and soluble BAK^13,33,42^ (ΔN22/ΔC25, obtained by deleting the N-terminal residues 1-22 and the transmembrane domain residues 187-211 from full-length BAK), which is used throughout this manuscript. We investigated and validated the Parkin-mediated Ub modification at BAK K113 in vitro. By engineering a mimetic of K113-ubiquitinated BAK^33^, disulfide–linked Ub^G76C^ ~ BAK^K113C^, we generate a NMR-derived structure model of the K113-ubiquitinated BAK complex. The L8-I44-H68-V70 hydrophobic patch of the conjugated Ub subunit binds to the canonical hydrophobic groove of BAK. The binding of Ub in the BAK groove occludes the binding of prodeath BH3 activators and impairs BID-induced BAK activation and membrane permeabilization. Point mutations targeting the BAK-associating interface in the Ub subunit dissociate Ub from the BAK hydrophobic groove, allowing BID to activate this ubiquitinated BAK. Our findings provide structural and mechanistic insights into the inhibition of BAK apoptotic function by Parkin-mediated Ub modification.

## Results

### Fully activated Parkin can ubiquitinate truncated and soluble BAK at K113 in vitro

Previous cell-based assays showed that BAK ubiquitination occurred in cells expressing wild-type Parkin but was impaired in cells expressing loss-of-function mutants^33^. To directly characterize BAK targeting by Parkin and to validate the previous cellular results showing that BAK could be ubiquitinated at K113^33^, we performed an in vitro ubiquitination assay. Parkin is activated during mitophagy, in which its full E3 activity is stimulated by PINK1-mediated phosphorylation^43–45^ and the binding of PINK1-phosphorylated ubiquitin (pUb)^46^. Here we prepared phosphorylated human Parkin (pParkin) and assessed its ability to ubiquitinate BAK in the presence of pUb (Methods). To perform the ubiquitination assay and the following structure and mechanism studies in our work, we used a truncated, soluble human recombinant BAK (ΔN22/ΔC25, obtained by deleting the N-terminal residues 1-22 and transmembrane domain residues 187-211 from full-length BAK)^13,33,42^. The Resuls showed that BAK was modified with an Ub moiety in vitro, whereas little ubiquitination was detected on K113A-substituted BAK (Supplementary Figure 1). Polyubiquitin (Ub_2_, Ub_3_ and Ub_n_) and auto-ubiquitinated pParkin (pParkin-Ub_n_) were also generated (Supplementary Figure 1), as observed in ubiquitination assays of other Parkin substrates^47,48^. K113 in the truncated BAK is the only lysine available for modification by Parkin (as the second lysine, K210, is deleted). This is consistent with our observation that the truncated BAK is only mono-ubiquitinated. Our biochemical results therefore verify previous cellular results that BAK is predominantly mono-ubiquitinated by Parkin at K113 during mitophagy^33^. Since the second lysine, K210, is absent in our truncated BAK construct, we cannot exclude the possibility that Parkin may also modify Ub at this site, which has been proposed to undergo ubiquitination following the formation of BAK oligomer pores^33^.

### Classical L8-I44-H68-V70 hydrophobic patch in Ub subunit interacts with BAK hydrophobic groove in K113-ubiquitinated BAK

**Fig. 1:**
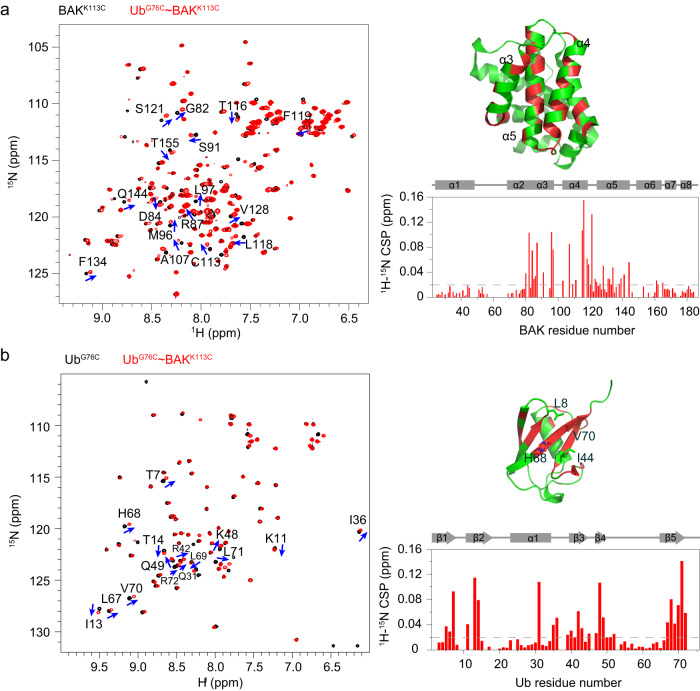
The K113-conjugated Ub subunit employs its classical L8-I44-H68-V70 hydrophobic patch to interact with the hydrophobic groove of BAK. **a** Chemical shift perturbations (CSPs) of BAK upon Ub conjugation. Left, superimposed ^1^H-^15^N HSQC spectra of ^15^N-labeled BAK^K113C^ (black) and Ub^G76C^ ~ BAK^K113C^ mimetic (red). Right, the ^1^H-^15^N CSPs of BAK upon K113-Ub conjugation are plotted against BAK residue number. The secondary elements of BAK are labeled at the top of the CSPs plot. Residues harboring large ^1^H-^15^N CSPs are highlighted using blue arrows in the ^1^H-^15^N HSQC spectrum (>0.04 ppm) and mapped on the BAK structure (2IMT.PDB^42^) in red (>0.02 ppm). The α3-α5 helices constituting the hydrophobic groove of BAK are indicated on its structure. **b** CSPs of Ub conjugated to BAK. Left, superimposed ^1^H-^15^N HSQC spectra of ^15^N-labeled Ub^G76C^ (black) and Ub^G76C^ ~ BAK^K113C^ mimetic (red). Right, The ^1^H-^15^N CSPs of Ub upon linking to BAK are plotted against Ub residue number. The secondary elements of Ub are labeled at the top of the CSPs plot. Residues harboring large ^1^H-^15^N CSPs are highlighted using blue arrows (>0.04 ppm) and mapped on the Ub structure (1UBQ.PDB^76^) in red (>0.02 ppm). Residues of the L8-I44-H68-V70 hydrophobic patch are shown in stick representation. The ^1^H-^15^N CSP is calculated as (0.5 × ΔH^2^ + 0.1 × ΔN^2^)^0.5^, where ΔH and ΔN stand for the changed chemical shift values in the proton and nitrogen dimensions, respectively. The gray dashed line indicates a CSP of 0.02 ppm.

We next constructed a previously used functional mimetic of K113-ubiquitinated BAK^33^, Ub^G76C^ ~ BAK^K113C^, for inhibition mechanism studies. Ub^G76C^ ~ BAK^K113C^ was generated by specific disulfide–linking between a Ub construct carrying the C-terminal G76C substitution (Ub^G76C^) and truncated BAK (BAK^K113C^, ΔN22/ΔC25 and carrying C166S and K113C substitutions)^33,49^. The strategy of disulfide engineering has been broadly used in characterizing protein complexes and interactions^50–52^. Using this mimetic, we initially aimed to assess interaction between the two subunits in K113-ubiquitinated BAK. We separately prepared ^15^N-labeled BAK^K113C^ and Ub^G76C^ ~ BAK^K113C^ with ^15^N-labeled BAK^K113C^ subunit and unlabeled Ub^G76C^ subunit. By NMR spectroscopy, ^1^H-^15^N HSQC spectra of these two samples were recorded to monitor chemical shift perturbations (CSPs) of BAK upon Ub conjugation (Fig. 1a). Consequently, conspicuous shifts of a subset of cross peaks were observed, corresponding to residues located mainly in the hydrophobic groove (α3-α5 region) (Fig. 1a). These BAK CSPs induced by Ub conjugation thus suggest that the covalently linked Ub interacts with and binds within the hydrophobic groove of BAK.

In parallel, the interface on the Ub subunit of Ub^G76C^ ~ BAK^K113C^ that interacts with the BAK subunit was further determined. A comparison between chemical shift values for Ub^G76C^ and those for Ub^G76C^ ~ BAK^K113C^ showed movement of some cross peaks (Fig. 1b), again indicating the existence of interaction between the two subunits in the covalently linked complex. The Ub residues that show large CSPs upon linking to BAK are located mainly in Ub β strands and include T7, K11, I13, T14, Q31, I36, R42, K48, Q49, L67, H68, L69, V70, L71 and R72 (Fig. 1b). These residues form a continuous interface that spans the L8-I44-H68-V70 hydrophobic patch in the β-sheet of ubiquitin, a classical surface serving as the platform for interaction with hundreds of ubiquitin-binding domain-containing proteins^53^. Together, our results indicate that in K113-ubiquitinated BAK, the classical hydrophobic patch of the Ub subunit interacts with the hydrophobic groove of the BAK subunit.

### The solution structure model of the K113-ubiquitinated BAK complex

To gain more insights into the inhibition of BAK activity by K113-Ub modification, we further sought to determine the structure model of Ub^G76C^ ~ BAK^K113C^ using NMR methods. The 3D F_1_-^15^N/^13^C filtered, F_2_-^13^C edited NOESY of Ub^G76C^ ~ BAK^K113C^ (^13^C/^15^N-labeled BAK^K113C^ subunit and unlabeled Ub^G76C^ subunit) was acquired to explore the details of the interaction between BAK and Ub subunits. Several strips of cross-peaks were observed (Supplementary Figure 2), and they were assigned via previously published chemical shift values of BAK^13^, Ub^54^ and our additional 3D ^15^N-edited NOESY spectra (Methods). The observed inter-subunit nuclear Overhauser effects (NOEs) show that the methyl groups of the Ub V70 residue are in close contact with the methyl groups of M96 and I114 in BAK (Supplementary Figure 2). In addition, the NOESY spectrum indicates that the side chain of the Ub R42 residue is also involved in the interaction with BAK (Supplementary Fig. 2). Based on the intensities of cross peaks, the observed inter-subunit NOEs were used to derive distance restraints, which were divided into three ranges for structure calculation: short-range (2.8 Å), medium-range (3.2–3.5 Å) and long-range (3.8–4.2 Å) (Supplementary Table 1). Taking these NOE-derived distance restraints (Supplementary Table 1), the structure of Ub^G76C^ ~ BAK^K113C^ was calculated using Xplor-NIH^55^. For the structure calculation, rigid body refinement was performed, with torsion angle freedom given to the linker between the Ub C-terminus loop (including residues 72-76) and the side chain of BAK K113C disulfide–linked to Ub G76C. In addition to the NOE-derived distance restraints, covalent energy terms (including bonds, angles and impropers) and van der Waals repulsive terms were also employed in the calculation. During the simulated annealing calculation, the temperature was decreased from 3000 °C to 25 °C with a temperature step of 12.5 °C. In total, 240 structure models of Ub^G76C^ ~ BAK^K113C^ were calculated. An ensemble of the resultant 20 lowest-energy structure models exhibited converged conformations, with a backbone-atom RMSD of 0.91 ± 0.30 Å (Supplementary Fig. 3), and no NOE violation (>0.5 Å) was observed for these structure models (Table 1). The lowest-energy conformer was used for further analysis and is shown in the manuscript.

**Table 1:** The structure statistics of the 20 Ub^G76C^ ~ BAK^K113C^ structures with lowest energies.

Distance restraints	
Number of NOE restraints	14
Violation (>0.5 Å)	0
RMS	0.19 ± 0.03
RMS from idealized geometry	
Bonds (Å)	0.004 ± 0.0008
Angles (°)	0.49 ± 0.04
Impropers (°)	0.38 ± 0.03
Average Pairwise r.m.s. deviation (Å)	
All atoms	1.46 ± 0.19
Back-bone atoms	0.91 ± 0.30
Ramachandran statistics (%)	
Residues in most favored regions	82.0
Residues in additional allowed regions	16.3
Residues in generously allowed regions	0.5
Residues in disallowed regions	1.1

The 20 conformers with the lowest energies were selected for statistical analysis. The pairwise RMSD was calculated with aligned BAK subunit. The Ramachandran statistics are based on PROCHECK-NMR analysis.

**Fig. 2:**
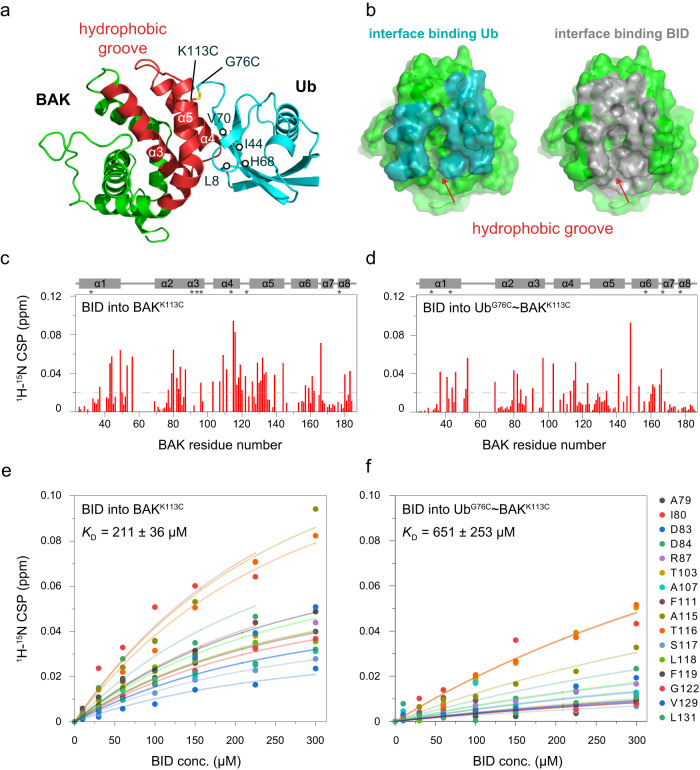
The solution structure of Ub^G76C^ ~ BAK^K113C^, showing that the Ub harbored in the BAK hydrophobic groove occludes BID binding. **a** The resolved NMR structure model of Ub^G76C^ ~ BAK^K113C^. Subunits are shown in cartoon representation. The α3-α5 helices of the BAK subunit are highlighted and the assembled hydrophobic groove is colored in red. The L8-I44-H68-V70 hydrophobic patch of the Ub subunit is also marked. The disulfide bond between Ub^G76C^ and BAK^K113C^ is shown as a stick. **b** The binding surface of BAK for the Ub subunit (cyan) overlaps with that for BID (gray). Red arrows indicate the BAK hydrophobic groove. The previously reported BAK/BID complex structure (2M5B.PDB)^13^ is used here for analysis. **c**, **d** BID titration-induced CSPs of BAK and Ub^G76C^ ~ BAK^K113C^, respectively. The ^1^H-^15^N CSPs of 100 μM ^15^N-labeled BAK^K113C^ or 100 μM Ub^G76C^ ~ BAK^K113C^ (with ^15^N-labeled BAK^K113C^ subunit) upon the addition of 300 μM BID BH3 peptide are plotted against BAK residue number. The secondary elements of BAK are labeled at the top of the CSPs plot, and the signals that disappeared during titration are indicated using asterisks. The gray dashed line indicates a CSP of 0.02 ppm. **e**, **f** *K*_D_ determinations of the BAK^K113C^/BID interaction and Ub^G76C^ ~ BAK^K113C^/BID interaction, respectively. The BID titration-induced ^1^H-^15^N CSPs of BAK hydrophobic groove residues are globally fitted to the titrated BID concentrations. The residues involved in the fitting are shown on the right side. The ^1^H-^15^N CSP is calculated as (0.5 × ΔH^2^ + 0.1 × ΔN^2^)^0.5^, where ΔH and ΔN stand for the changed chemical shift values in the proton and nitrogen dimensions, respectively.

Ub^G76C^ ~ BAK^K113C^ adopts a compact conformation, wherein the BAK and Ub subunits are associated (Fig. 2a). The buried solvent accessible surface area (SASA) between the two subunits is ~1323 Å^2^. The hydrophobic groove of BAK, assembled by α3-α5 helices, is attached to the L8-I44-H68-V70 hydrophobic patch in the Ub β-sheet region (Fig. 2a). This conformation is consistent with our identified interface between the Ub and BAK subunits, monitored by ^1^H-^15^N HSQC spectra (Fig. 1).

### Ub modification at BAK K113 occludes the hydrophobic groove and prevents BID binding

The Ub^G76C^ ~ BAK^K113C^ structure model provides a framework for understanding how the Ub modification at BAK K113 inhibits BH3 binding-induced BAK activation. BAK hydrophobic groove is the conserved binding site for BH3 activators^11,13,21,56^ (Supplementary Figure 4). Superposing the Ub^G76C^ ~ BAK^K113C^ structure and the reported structure of the BAK/BID complex^13^ using BAK as a reference shows that the position of K113-conjugated Ub overlaps well with that of the associated BID (Fig. 2b and Supplementary Figure 5). Consequently, the K113-conjugated Ub occludes BAK hydrophobic groove and produces steric clashes with BID, which would most likely prevent BID binding.

To further corroborate this structural finding, we examined the binding affinities of the BID BH3 peptide bound to BAK and Ub^G76C^ ~ BAK^K113C^. Titrating the BID BH3 peptide into ^15^N-labeled BAK^K113C^ or Ub^G76C^ ~ BAK^K113C^ (^15^N-labeled BAK^K113C^ subunit and unlabeled Ub^G76C^ subunit) caused a subset of residues to shift progressively or disappear (Fig. 2c, d and Supplementary Fig. 6). The perturbed residues are located mainly in the BAK hydrophobic groove (α3-α5 helices) and the two CSP profiles are similar, whereas smaller CSPs are observed in BID/Ub^G76C^ ~ BAK^K113C^ titration (Fig. 2c, d). These results indicate that BID BH3 peptide binds to the same surface on BAK and on K113-ubiquitinated BAK. The detectable CSPs (>0.02 ppm) of hydrophobic groove residues collected in BID/BAK titration were further used to globally fit the binding affinity between BAK and the BID BH3 peptide, affording a dissociation constant (*K*_D_) of 211 ± 36 μM (Fig. 2e). This NMR-measured *K*_D_ value is comparable to that previously reported using other methods^13,21^, despite being much weaker than the binding of a known “stapled” helical version of BID BH3 peptide (*K*_D_ ≈ 1 μM) which results in large BAK CSPs reaching up to 1.0 ppm^13^. In parallel, the CSPs of these hydrophobic groove residues in the BID/Ub^G76C^ ~ BAK^K113C^ titration were also used to fit the *K*_D_ of their interaction (Fig. 2f). A *K*_D_ of approximately 651 ± 253 μM was obtained for ubiquitinated BAK although the CSPs are small (Fig. 2f), revealing that Ub modification of BAK reduces its BID-binding ability by approximately 3-fold.

Our structural characterization and interaction analysis collectively demonstrate that K113 ubiquitination of BAK impairs BID binding, by occupying the BID binding site in the BAK hydrophobic groove and therefore explains why Ub modification can inhibit prodeath BH3-induced BAK activation^33^.

### Disrupting the interface of the Ub subunit reduces its association within the BAK hydrophobic groove and allows BID-induced apoptotic activity

**Fig. 3:**
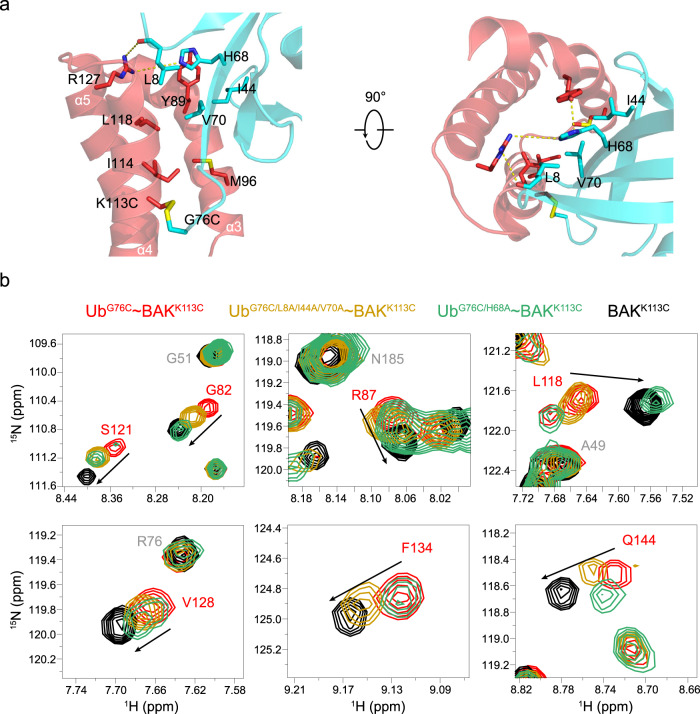
Interactions between the two subunits of the Ub^G76C^ ~ BAK^K113C^ complex and disrupting the interface of the Ub subunit reduces its association within the BAK hydrophobic groove. **a** The interaction interface between the two subunits. Residues involved in the interaction are shown as in stick representation. The α3-α5 helices of BAK that form the hydrophobic groove are colored in red. **b** Representative cross peaks of the BAK hydrophobic groove residues in ^1^H-^15^N HSQC spectra. The spectra of the ^15^N-labeled BAK subunit in Ub^G76C^ ~ BAK^K113C^, Ub^G76C/L8A/I44A/V70A^ ~ BAK^K113C^, Ub^G76C/H68A^ ~ BAK^K113C^ and BAK^K113C^ are colored red, orange, green and black, respectively. Black arrows indicate the movements of cross peaks.

Our solution structure model of Ub^G76C^ ~ BAK^K113C^ elucidates the mechanism underlying the blocking of the BAK hydrophobic groove by K113-ubiquitination. Hydrophobic interactions between the L8-I44-H68-V70 hydrophobic patch of K113-conjugated Ub and the hydrophobic groove of the BAK subunit assemble the closely associated conformation (Fig. 2a). Specifically, the methyl groups of L8, I44 and V70 of the Ub subunit point into the hydrophobic groove between the α3-α5 helices of BAK, and these methyl groups of Ub participate in hydrophobic interactions with Y89, M96, I114 and L118 of BAK (Fig. 3a). In addition, the side chain of H68 in Ub likely interacts with Y89 and R127 of BAK helping to hold the two subunits together (Fig. 3a). Consequently, BAK hydrophobic groove is blocked by K113-conjugated ubiquitin.

To further assess whether disrupting the BAK-associating interface in the Ub subunit would unlock the hydrophobic groove of BAK, we generated two mutant Ub^G76C^ ~ BAK^K113C^ samples with a ^15^N-labeled BAK subunit for NMR analysis. One contains the Ub L8A/I44A/V70A triple mutation, and the other contains the Ub H68A single point mutation (termed Ub^G76C/L8A/I44A/V70A^ ~ BAK^K113C^ and Ub^G76C/H68A^ ~ BAK^K113C^, respectively). The ^1^H-^15^N HSQC spectra comparison between BAK^K113C^ and Ub^G76C^ ~ BAK^K113C^ wild type reveals that some cross peaks of residues located at the BAK hydrophobic groove such as G82 and R87 in α3, L118 and S121 in α4, and V128, F134 and Q144 in α5 are shifted by Ub conjugation (Fig. 3b). These perturbed residues indicate that the Ub subunit is harbored at the hydrophobic groove of BAK. Introducing Ub point mutations and obtaining ^1^H-^15^N HSQC spectra of Ub^G76C/L8A/I44A/V70A^ ~ BAK^K113C^ and Ub^G76C/H68A^ ~ BAK^K113C^, however, showed that the cross peaks of these hydrophobic groove residues were partially or completely restored to the positions in BAK^K113C^ (Fig. 3b), suggesting that the mutant Ub subunit associates less strongly than the wild type with the hydrophobic groove. Consistent with this NMR observation, small-angle X-ray scattering (SAXS) analysis reveals that the compact conformation of Ub^G76C^ ~ BAK^K113C^ becomes extended upon Ub subunit mutations (Supplementary Figure 7) owing to the attenuated association between BAK and mutant Ub subunits. We therefore speculate that the Ub interface mutations undo the blocking of BAK hydrophobic groove and thus expose it for BID activation.

**Fig. 4:**
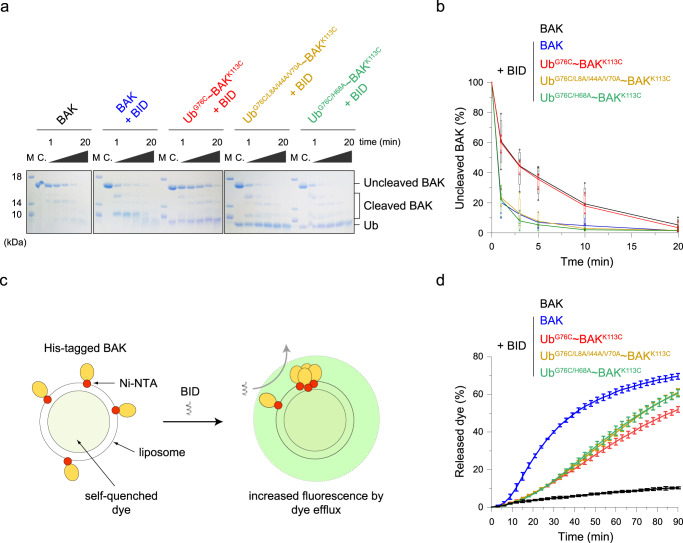
Reduced association of the Ub subunit within the BAK hydrophobic groove allows BID-induced apoptotic activity. Limited proteolysis assay of BID-induced BAK activation resolved on SDS-PAGE gel **a** and quantified **b**. C, the sample was not incubated with proteinase K and was used as a reference. The proteolysis reactions were stopped at given times (1, 3, 5, 10 and 20 minutes), and the product mixture was treated with 10 mM DTT to reduce the disulfide–linked complex before transferring to the SDS-PAGE gel. The experiments were repeated four times independently with similar results. The SDS-PAGE gel bands were quantified using ImageJ and were averaged from four independent repeats, and the error bar indicates the SD. **c** Schematic design of the fluorescence-based liposome assay and **d** time-course of dye efflux by monitoring fluorescence increase. One-bench prepared liposome was used for all the samples and these experiments were completed within 10 hours. The data from three independent measurements are averaged, and the error bar indicates the SD.

To clarify whether the reduced association between Ub and BAK subunits described above may allow the BID BH3 peptide to reactivate ubiquitinated BAK, we further performed limited proteolysis, an assay monitoring BID-induced BAK activation, wherein the surface of activated BAK is exposed and becomes susceptible to proteolysis^15,49,57^. Our time-course results of proteinase K digestion experiments showed that the presence of the BID BH3 peptide causes BAK to cleave faster with limited proteolysis, whereas K113-Ub conjugation increases BAK resistance (Fig. 4a, b), consistent with previous findings that ubiquitination inhibits BID-induced BAK activation^33^. Incubating with BID showed that the Ub^G76C/L8A/I44A/V70A^ ~ BAK^K113C^ and Ub^G76C/H68A^ ~ BAK^K113C^ mutants are more sensitive to proteinase than Ub^G76C^ ~ BAK^K113C^ wild type and are efficiently cleaved, similar to nonubiquitinated BAK (Fig. 4a, b). Therefore, the relaxed Ub association within the BAK hydrophobic groove, induced by Ub L8A/I44A/V70A or H68A mutation, impairs Ub-induced inhibition and allows BID to activate ubiquitinated BAK.

We further performed a liposome dye release assay to analyze the effect of the attenuated Ub association on BAK apoptotic function. The minimal model liposome system has been effectively used to represent BID-activated BAK oligomerization and membrane permeabilization in studies of apoptotic cell death^9,33,56,58^. This is achieved by targeting His-tagged BAK to liposome though Ni-NTA lipids (Fig. 4c). The liposome mimics the mitochondrial outer membrane by constructing a mixed lipid composition, as in the mitochondrial outer membrane, which allows BID BH3 peptide to bind the hydrophobic groove of the liposome-targeted BAK and subsequently activate BAK to oligomerize and permeabilize liposome membrane (Fig. 4c). As a result, increased fluorescence is observed owing to the efflux of self-quenched fluorescent dye pre-encapsulated in the liposome (Fig. 4c). We found that the liposome permeabilization activity of BAK was increased upon activation with BID BH3 peptide, whereas it was largely inhibited by K113-Ub conjugation (Fig. 4d), in line with previous reports^20,24,33,56,58^. For Ub L8A/I44A/V70A and H68A mutants, which impair the ability of Ub to block the BID-binding site of BAK (Fig. 3b), we observed increased liposome permeabilization kinetics of the Ub^G76C/L8A/I44A/V70A^ ~ BAK^K113C^ and Ub^G76C/H68A^ ~ BAK^K113C^ mutants compared to Ub^G76C^ ~ BAK^K113C^ wild type (Fig. 4d). These results again suggest that Ub L8A/I44A/V70A and H68A mutations likely make the BAK hydrophobic groove more accessible to BID in ubiquitinated BAK. Collectively, our structural analyses and functional evidence reveal that Ub modification blocks the BID-binding site of BAK and thus inhibits BID-induced apoptotic activity. Additionally, point mutations targeting the BAK-binding interface of Ub dissociate Ub from BAK, thereby exposing the BAK hydrophobic groove for BID activation.

## Discussion

Here, we present a solution structure model of the K113-ubiquitinated BAK complex that adopts a compact conformation (Fig. 2a). Considering the extensive binding surface between the Ub and BAK subunits (Fig. 2a) which overlaps the region of BAK that participates in BID binding (Fig. 2b), significant CSPs are expected in each subunit upon inter-subunit association, in addition to a greatly reduced binding affinity of BAK for BID. However, small association-induced CSPs are observed (Fig. 1), and Ub modification of BAK only moderately reduces its BID-binding ability by approximately 3-fold (Fig. 2e, f). What is the reason for the contrasts between structural implication and experimental observations? We speculate that conjugated Ub may be weakly and potentially transiently bound to BAK and in equilibrium with co-existing compact (closed) and extended (open) forms, wherein the closed state is preferred in solution and thus was captured here. The equilibrium between these two states would account for the relatively small change in BID binding affinity, since the BID binding site would be occupied only part of the time. This transient and equilibrium mechanism may also explain the minor difference observed between the Ub^G76C^ ~ BAK^K113C^ and Ub^G76C^ ~ BAK^K113C^ mutants in their ability to bind the BID peptide or alter membrane permeabilization (Fig. 4). Transient inter-subunit interactions and the model of equilibrium between closed and open states in Ub-conjugated protein complexes have been well established^59–66^.

Protein ubiquitination is a ubiquitous form of post-translational modification. Conjugated Ub functions as a versatile signal that controls almost every cellular process by providing binding sites for many Ub-binding proteins^67,68^. In the case of Parkin-mediated BAK ubiquitination, conjugated Ub acts as a blocker, occluding the prodeath BH3-binding site, thereby suppressing BH3-stimulated BAK apoptotic activity. Therefore, our findings now suggest a nonsignaling function of Ub in directly antagonizing activators targeting Ub-modified proteins rather than by recruiting downstream partners for cellular messaging.

Prodeath BH3 activators employ a hit-and-run mechanism to stimulate the apoptotic activity of BAK and BAX effectors^8,69^. BH3s weakly bind to the canonical hydrophobic groove of these effectors and then dissociate^12–17^. This transient binding of BH3s triggers subsequent conformational changes in these apoptotic effectors and ultimately results in pore-forming homo-oligomers^18–24^. Previous NMR experiments showed that BH3s binding within the hydrophobic groove of BAK and BAX (α3-α5 region) could induce additional CSPs mapping to residues on either side of the groove in helices α1 and α6-α8^13,22,23^. These perturbations are topologically opposite to the BH3-binding site and are further thought to be the initial conformational changes needed for BAK and BAX activation^13,20,21,24^. Here, we found that while BID BH3 peptide binding induces conspicuous CSPs in the BAK α1 and α6-α8 helices (Fig. 2c), few CSPs are observed in this region upon Ub conjugation and association (Fig. 1a). Therefore, we speculate that the ubiquitination-inhibited BAK activity might be attributable not only to the blocking of BID binding by conjugated Ub but also to the resultant suppression of the dynamics of helices α1 and α6-α8. It has been proposed and becomes clear that Ub signaling also depends on the modified protein undergoing Ub or UBL (Ub-like protein)-dependent conformational changes^70,71^. For example, UBL NEDD8 modification of cullin–RING ligases (CRLs) allosterically stimulates CRLs structural changes and promotes CRLs activity, affecting outcomes^72–75^. The implication of our observation of Ub-suppressed BAK dynamics might provide another example of Ub modification further limiting the behavior of the modified protein. Future dynamics studies of ubiquitinated BAK would provide insights into this aspect.

## Methods

### Protein expression and purification

For the preparation of BAK, the codon-optimized DNA of human BAK (ΔN22 ΔC25 C166S K113C) was cloned into pET15 vector with a N-terminal 6 × His-tag following a DrICE protease cleavage site, and expressed in *E. coli* strain BL21(DE3) using Lysogeny broth (LB) medium. The cells were induced with 0.2 mM isopropyl-β-D-thiogalactoside (IPTG) at 16 °C for 14-16 hours. For NMR sample preparation, cells were cultured in M9 medium, using ^15^N NH_4_Cl and ^13^C glucose as the sole source of nitrogen and carbon, respectively. The harvest cell pellets were resuspended in a buffer containing 25 mM Tris-HCl (pH 8.0), 150 mM NaCl and lysed by high-pressure cell disrupter. Target protein was collected from the supernatant and purified over Ni^2+^ affinity resin. The 6 × His-tag of BAK was removed by DrICE digestion and further purified using HiTrap Q anion exchange column (GE Healthcare) and size-exclusion chromatography (Superdex75 Increase 10/300, GE Healthcare) in tandem. BAK was prepared in a buffer containing 25 mM Tris-HCl (pH 8.0), 150 mM NaCl for further use. BAK mutants were constructed using Gibson Assembly method, verified by DNA sequencing and prepared in the same way as the wild type.

The DNA of human Ub was cloned into pET11 vector and expressed as above described for BAK. Purification was sequentially performed over SP-FF column (GE Healthcare), HiTrap S cation exchange column (GE Healthcare) and size-exclusion chromatography (Superdex75 Increase 10/300, GE Healthcare). Purified Ub was prepared in a buffer containing 25 mM Tris-HCl (pH 8.0), 150 mM NaCl for further use. ^15^N- and ^13^C-labeld Ub were expressed using M9 medium. Ub mutants were constructed using Gibson Assembly method, verified by DNA sequencing and prepared in the same way as the wild type.

Human GST-fused Uba1 was tagged with a N-terminal 6 × His-tag and expressed in *E. coli* strain BL21(DE3). Cells were induced with 0.2 mM IPTG at 18 °C for 14-16 hours. Target protein was purified over Ni^2+^ affinity resin and HiTrap Q anion exchange column (GE Healthcare), and was prepared in a buffer containing 25 mM Tris-HCl (pH 7.5) and 150 mM NaCl.

The DNA of human UbcH7 was cloned into pET11 vector and expressed in *E. coli* strain BL21(DE3). Cells were induced with 0.2 mM IPTG at 18 °C for 12 hours. Target protein was purified using SP-FF column (GE Healthcare) and was prepared in a buffer containing 25 mM Tris-HCl (pH 7.5), 150 mM NaCl.

The DNA of human Parkin was cloned into a modified pFastBac1 vector carrying a N-terminal 6×His-tag and a DrICE protease cleavage site. Protein was expressed in *Spodoptera frugiperda Sf9* cells at 27 °C for 60-65 hours after baculoviruse infection. Protein was purified over Ni^2+^ affinity resin and HiTrap Q anion exchange column (GE Healthcare), and was prepared in a buffer containing 25 mM Tris-HCl (pH 7.5) and 150 mM NaCl.

The DNA of *pediculus humanu* PINK1 (including residues 115-575) was cloned into PGEX-4T vector carrying a N-terminal GST-tag and expressed in *E. coli* strain BL21(DE3). Cells were induced with 0.2 mM IPTG at 16 °C for 14-16 hours. Target protein was purified using GST-4b resin (GE Healthcare) and was prepared in a buffer containing 25 mM Tris-HCl (pH 7.5), 150 mM NaCl.

### Preparation of phosphorylated Ub and phosphorylated Parkin

PINK1 phosphorylates Ub at S65 site^46^. For the preparation of S65 phosphorylated Ub (pUb), 3 μM PINK1 was incubated with 200 μM Ub in the buffer containing 25 mM Tris-HCl (pH7.5), 150 mM NaCl, 10 mM ATP and 15 mM MgCl_2_ at 16 °C for 9 hours. The pUb was purified using HiTrap Q anion exchange column (GE Healthcare) and size-exclusion chromatography (Superdex75 Increase 10/300, GE Healthcare), and prepared in a buffer containing 25 mM Tris-HCl (pH7.5) and 150 mM NaCl.

PINK1 phosphorylates Parkin at S65 site^43^. For the preparation of S65 phosphorylated Parkin (pParkin), 1 μM PINK1 was incubated with 25 μM Parkin in the buffer containing 25 mM Tris-HCl (pH7.5), 150 mM NaCl, 10 mM ATP and 15 mM MgCl_2_ at 16 °C for 5 hours. The pParkin was purified using HiTrap Q anion exchange column (GE Healthcare) and size-exclusion chromatography (Superdex75 Increase 10/300, GE Healthcare), and prepared in a buffer containing 25 mM Tris-HCl (pH7.5) and 150 mM NaCl.

### Ubiquitination assay

Reactions were performed at 30 °C by incubating 10 μM BAK, 60 μM Ub, 400 nM Uba1, 500 nM Ubch7, 3 μM pParkin, 3 μM pUb, 10 mM ATP and 15 mM MgCl_2._ in the buffer containing 25 mM Tris-HCl (pH 7.5), 150 mM NaCl. Reactions were stopped at given times (0, 30, 60, 90 and 120 minutes) by adding protein loading buffer, resolved by SDS-PAGE, and visualized using Coomassie-blue staining.

### Disulphide-linking of Ub^G76C^ ~ BAK^K113C^ mimetic

300 μM Ub^G76C^ and 60 μM BAK^K113C^ were incubated at room temperature in the presence of 0.08% H_2_O_2_ for 60 minutes. The specifically disulfide–linked Ub^G76C^ ~ BAK^K113C^ was further purified by using anion exchange chromatography (HiTrap Q, GE Healthcare) and size-exclusion chromatography (Superdex75 Increase 10/300, GE Healthcare). Targets were prepared in a buffer containing 25 mM Tris-HCl (pH 8.0), 150 mM NaCl. For the sample used in the liposome dye release assay, a 6 × His-tag was constructed at the C-terminus of BAK^K113C^. Ub^G76C^ ~ BAK^K113C^ mutants were prepared in the same way.

### NMR Spectroscopy

NMR experiments were recorded at 310 K using Bruker 600 MHz and Bruker 850 MHz spectrometers equipped with cryogenic probes. The NMR samples were prepared in sodium phosphate buffer (NaH_2_PO_4_/Na_2_HPO_4_ 20 mM, NaCl 100 mM, pH 6.8 and D_2_O 10%). To confirm the chemical shift assignment of the BAK subunit, CBCACONH and HNCACB spectra were acquired for the Ub^G76C^ ~ BAK^K113C^ sample (^13^C/^15^N-labeled BAK^K113C^ subunit and unlabeled Ub^G76C^ subunit). The three dimensional ^13^C-filtered NOESY of the Ub^G76C^ ~ BAK^K113C^ (^13^C/^15^N-labeled BAK^K113C^ subunit and unlabeled Ub^G76C^ subunit) was recorded to extract the inter-subunit NOEs. The cross peaks in the ^13^C-filtered NOESY were assigned based on 3D ^15^N-edited NOESY experiments of the Ub^G76C^ ~ BAK^K113C^ sample (^15^N-labeled BAK^K113C^ subunit and unlabeled Ub^G76C^ subunit) and the Ub^G76C^ ~ BAK^K113C^ sample (unlabeled BAK^K113C^ subunit and ^15^N-labeled Ub^G76C^ subunit), and according to the published data^13,54^. All the NMR data were processed using NMRPipe (Version 2020) and analyzed using CcpNmr analysis (Version 2.4.2).

The NMR titration experiments were conducted as addition of the concentration gradient of BID BH3 peptide (0 μM, 10 μM, 30 μM, 60 μM, 100 μM, 150 μM, 225 μM, 300 μM) into 100 μM ^15^N-labelel BAK^K113C^ or 100 μM Ub^G76C^ ~ BAK^K113C^ sample (^15^N-labeled BAK^K113C^ subunit and unlabeled Ub^G76C^ subunit). The changes in chemical shift values during titration were monitored using a series of ^1^H-^15^N HSQC spectra. The amide chemical shift perturbation was calculated as Δδ = (0.5 × (ΔH)^2^ + 0.1 × (ΔN)^2^)^1/2^, where ΔH and ΔN are the chemical shift difference in the proton and nitrogen dimensions, respectively.

### Assignment of the inter-subunit NOEs in Ub^G76C^ ~ BAK^K113C^

Initially, the chemical shifts (^1^H_f2_, ^13^C_f3_) of the inter-subunit NOE peaks observed in the ^13^C-filtered NOESY (^13^C/^15^N-labeled BAK^K113C^ subunit and unlabeled Ub^G76C^ subunit) were compared with the published chemical shifts^13^, aiming to obtain possible assignment of the CH groups in BAK subunit. Because of the Ub attachment, the observed chemical shifts of our Ub^G76C^ ~ BAK^K113C^ sample showed some variations to the published NMR data of BAK^13^. To further verify the assignment, the assigned ^1^H-^15^N HSQC and the intra-molecular NOEs in our ^15^N-edited NOESY (^15^N-labeled BAK^K113C^ subunit and unlabeled Ub^G76C^ subunit) were analyzed. By navigating the assigned amide signal in ^1^H-^15^N HSQC to ^15^N-edited NOESY, the assignment of the amide groups (^1^H_f2_/^15^N_f3_ dimensions) in the ^15^N-edited NOESY could be clearly determined. Then, the chemical shift (^1^H_f1_) of the possible intra-molecular NOE peaks of a given residue was compared with the chemical shift (^1^H_f2_) of the inter-subunit NOEs. If the chemical shift of the intra-molecular NOE matches that of the inter-subunit NOE, the assignment could be confirmed.

After assigning BAK subunit in the ^13^C-filtered NOESY, the assignment of Ub subunit (^1^H_f1_ dimension) was conducted with similar method. Initially, the observed chemical shift (^1^H_f1_) of the NOE peaks in ^13^C-filtered NOESY (^13^C/^15^N-labeled BAK^K113C^ subunit and unlabeled Ub^G76C^ subunit) was compared with the published data^54^ to identify the possible assignment. Then, the assignment candidate was further verified using assigned ^1^H-^15^N HSQC and ^15^N-edited NOESY (unlabeled BAK^K113C^ subunit and ^15^N-labeled Ub^G76C^ subunit). Based on the assigned ^1^H-^15^N HSQC, a certain residue could be identified in the ^15^N-edited NOESY. The intra-molecular NOEs related to this residue were analyzed, and thier ^1^H_f1_ chemical shifts were compared to the corresponding assignment candidate of Ub subunit. If the chemical shifts of the intra-molecular and the inter-subunit NOE peaks well match with each other, the assignment of Ub subunit could be confirmed.

### Structure calculation of Ub^G76C^ ~ BAK^K113C^

The NOEs extracted from the ^13^C-filtered NOESY experiment were used as distance restraints to guide the structure calculation using Xplor-NIH^55^. During calculation, the BAK template (2M5B.PDB^13^) and the ubiquitin template (1UBQ.PDB^76^) were treated as rigid bodies except the C-terminal of loop (R72-G76C) of ubiquitin and the side chain of BAK K113C disulfide–linked to Ub G76C. A total of 240 structures were calculated, and 20 structures with smallest deviation from the mean structures were selected for structure presentation and analysis. The structure figures were generated using PyMOL (Verison 2.5.2; Schrödinge).

### Small-angle X-ray scattering (SAXS)

SAXS data were collected at the BL19U2 beamline of the Shanghai Synchrotron Radiation Facility (SSRF). 250 μM protein sample were separately prepared in a buffer containing 20 mM Hepes (pH 6.8) and 100 mM NaCl for SAXS measurements. For each measurement, 20 consecutive frames of 1-sec exposure were recorded and averaged, providing no difference between the first and the last frames. The background scattering was collected for the matching buffer and was subtracted from the protein scattering data. The SAXS experiment was performed at room temperature. The data was visualized and analyzed using the software package ATSAS^77^.

### Limited proteolysis assay1ub

40 μM BAK or disulfide–linked Ub^G76C^ ~ BAK^K113C^ was digested by 1.2 μM proteinase K at room temperature. 200 μM synthesized BID BH3 peptide (DIIRNIARHLAQVGDSMDRSIPPGLV) was added as requirement. The reaction was performed in a buffer containing 25 mM Tris-HCl (pH 8.0), 150 mM NaCl and 1% CHAPS, and stopped by the addition of 100 mM PMSF at given times (1, 3, 5, 10 and 20 minutes). The product mixtures were treated with 10 mM DTT to reduce the disulfide-linked complex, analyzed by SDS-PAGE, and quantified using ImageJ. The percentage of uncleaved BAK fraction was referenced to the same sample not incubated with proteinase K. Mutant proteins were assessed in the same way.

### Liposome dye release assay

The assay was performed as described^33^. In brief, the mitochondrial outer membrane-mimicked liposome containing 53% phosphatidylcholine (w/v), 28% phosphatidylethanolamine (w/v), 11% phosphatidylserine (w/v), 8% cardiolipin (w/v) and supplemented with 5% nickel chelating lipid (1,2-Dioleoyl-sn-Glycero-3-[N-(5-amino-1-carboxypentyl)iminodiacetic-acid)-succinyl] (w/v)) to capture His_6_-tagged BAK. The liposome stock (1 mg/mL) encapsulated 50 mM self-quenched 5(6)-carboxy-fluorescein (Sigma-Aldrich) and was stored at −20 °C. Before using, prepared liposomes were extruded through a 100 nm pore size membrane 20 times and passed over a PD10 column to remove excess dye. 100 μL extruded liposomes was diluted into 9.9 mL assay buffer containing 10 mM Hepes (pH 7.5) and 135 mM KCl, and incubated with either 50 nM His_6_-tagged BAK or disulfide–linked Ub^G76C^ ~ BAK^K113C^. 5 μM BID BH3 peptide was added as requirement. Fluorescence changes were monitored with a EnSpire Multimode Plate Reader suing λ_ex_ = 485 nm and λ_em_ = 515 nm, in a bottom-reading mode. At the end of measurement, 1% CHAPS (m/v) was added to determine the maximal dye fluorescence of the fully permeabilized liposomes. The dye released by protein was calculated as a percentage of CHAPS control. All assays were repeated independently three times. One-bench prepared liposome was used for all the samples and these experiments were completed within 10 hours. Assays for mutant proteins were performed in the same way.

### Statistics and reproducibility

No statistical analyses were conducted in this paper. Experiments related to liposome dye release assays (Fig. 4d) were performed three independent measurements. Experiments related to ubiquitination assays (Supplementary Figure 1) and limited proteolysis assays (Fig. 4b) were performed four independent measurements. Plotted data were averaged from these independent measurements, and the error bar indicates the SD.

### Reporting summary

Further information on research design is available in the Nature Portfolio Reporting Summary linked to this article.

### Supplementary information

Peer Review File

Supplementary Information

Description of Additional Supplementary Files

Supplementary Data 1

Reporting Summary

## Data Availability

The atomic coordinate of Ub^G76C^ ~ BAK^K113C^ has been deposited at PDB with the accession code 8IVB. Raw SDS-PAGE gel images of Fig. 4a and Supplementary Figure 1 are provided in Supplementary Figures 8 and 9. Source data for Figs. 4b and 4d are included in the Supplementary Data 1. Other data and materials are available from the corresponding authors on request.
