# Supplementary material for: Parkin-mediated ubiquitination inhibits BAK apoptotic activity by blocking its canonical hydrophobic groove
Source: Commun Biol. 2023 Dec 12;6:1260. doi: 10.1038/s42003-023-05650-z (PMC10716173; doi:10.1038/s42003-023-05650-z)
Supplement: Supplementary file 2 — Supplementary Information [file 42003_2023_5650_MOESM2_ESM.pdf]

Supplementary Information for

Parkin-mediated ubiquitination inhibits BAK apoptotic activity by blocking its canonical hydrophobic groove

Peng Cheng[1,#], Yuzhu Hou[1,#], Mingxing Bian[1], Xueru Fang[1], Yan Liu[1], Yuanfang Rao[1], Shuo Cao[1], Yanjun Liu[1], Shuai Zhang[2], Yanke Chen[2,*], Xu Dong[3,*,‡], Zhu Liu[1,*]

[#]These authors contributed equally to this work.

[‡]Current address: State Key Laboratory of Biocatalysis and Enzyme Engineering, College of Life Sciences, Hubei University, Wuhan 430074, China

[*]Corresponding author: yankec@mail.hzau.edu.cn (Y. C.), dongxu@wipm.ac.cn (X. D.), liuzhu@hzau.edu.cn (Z. L.).

This PDF file includes:

Supplementary Figures 1-9

Supplementary Tables 1

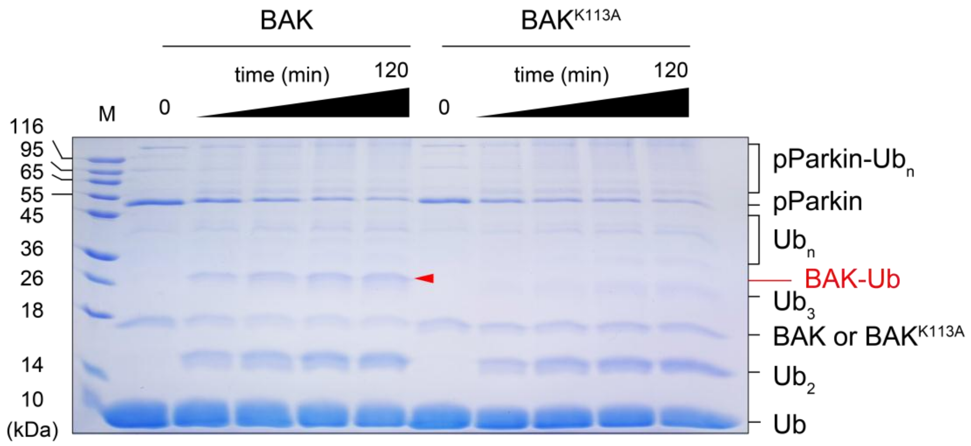

Supplementary Figure 1. *In vitro* assay for Parkin-mediated ubiquitination of the truncated and soluble BAK at K113. The ubiquitination reactions were stopped at given times (0, 30, 60, 90 and 120 minutes), resolved on SDS-PAGE gel, and visualized using Coomassie-blue staining. Ub_2, dimer ubiquitin; Ub_3, trimer ubiquitin; Ub_n, polyubiquitin; pParkin-Ub_n, auto-ubiquitinated pParkin; BAK-Ub, mono-ubiquitinated BAK. The red triangle indicates the mono-ubiquitinated BAK. The experiments were repeated four times independently with similar results.

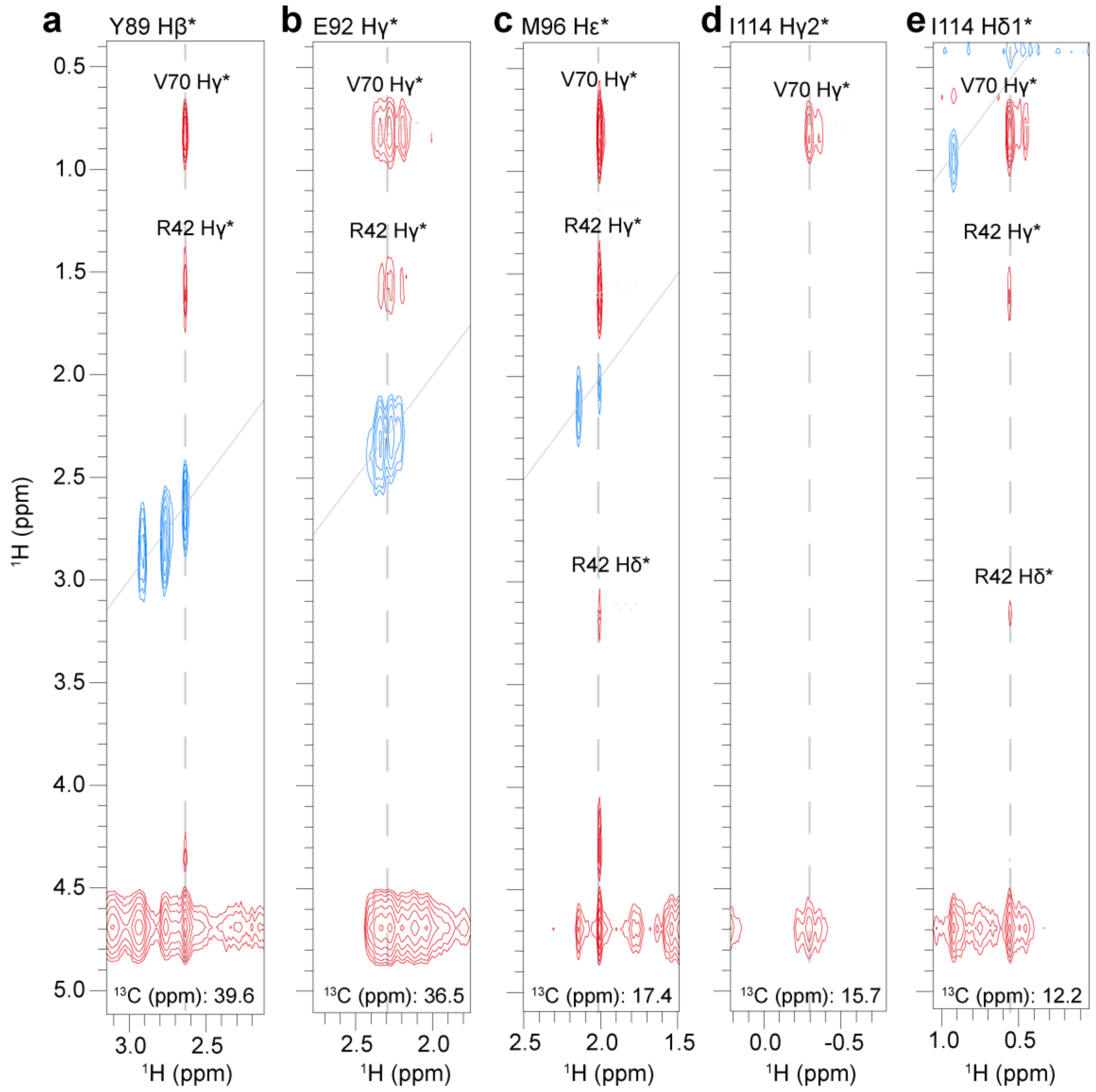

Supplementary Figure 2. Representative ^{1}H-^{1}H dimension plots of 3D filtered NOESY of Ub^{G76C}~BAK^{K113C} (^{13}C/^{15}N-labeled BAK^{K113C} subunit and unlabeled Ub^{G76C}). The peaks of BAK Y89 Hβ, BAK E92 Hγ, BAK M96 Hε, BAK I114 Hγ2 and BAK I114 Hδ1 from methyl groups are shown in panels (**a**), (**b**), (**c**), (**d**) and (**e**), respectively, and the ^{13}C chemical shift values are shown at the bottom of the panels. The peaks along the diagonal are shown in blue, and the cross peaks are shown in red. The assignments are indicated by the corresponding peaks.

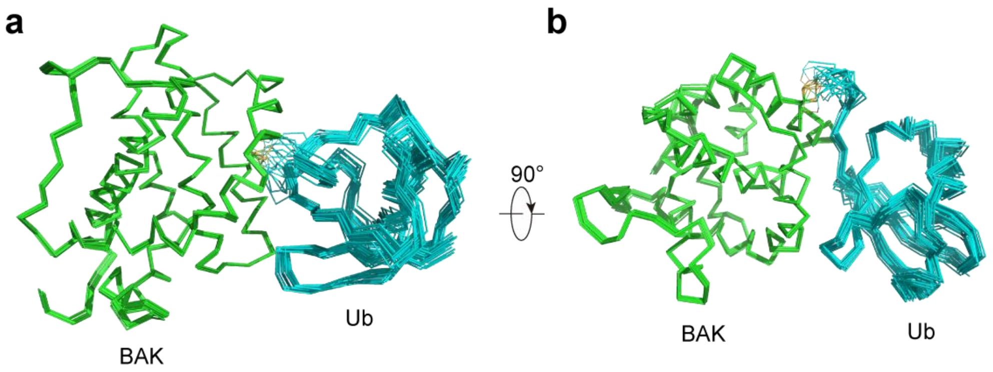

Supplementary Figure 3. The ensemble of 20 structures with lowest energies are shown as ribbon in two stereoscopic views. The BAK subunit and the Ub subunit are shown in green and cyan, respectively, and BAK K113C and Ub G76C residues are shown as sticks. All the structures are aligned with BAK subunit. The RMS deviation of all backbone atoms is 0.91±0.30 Å.

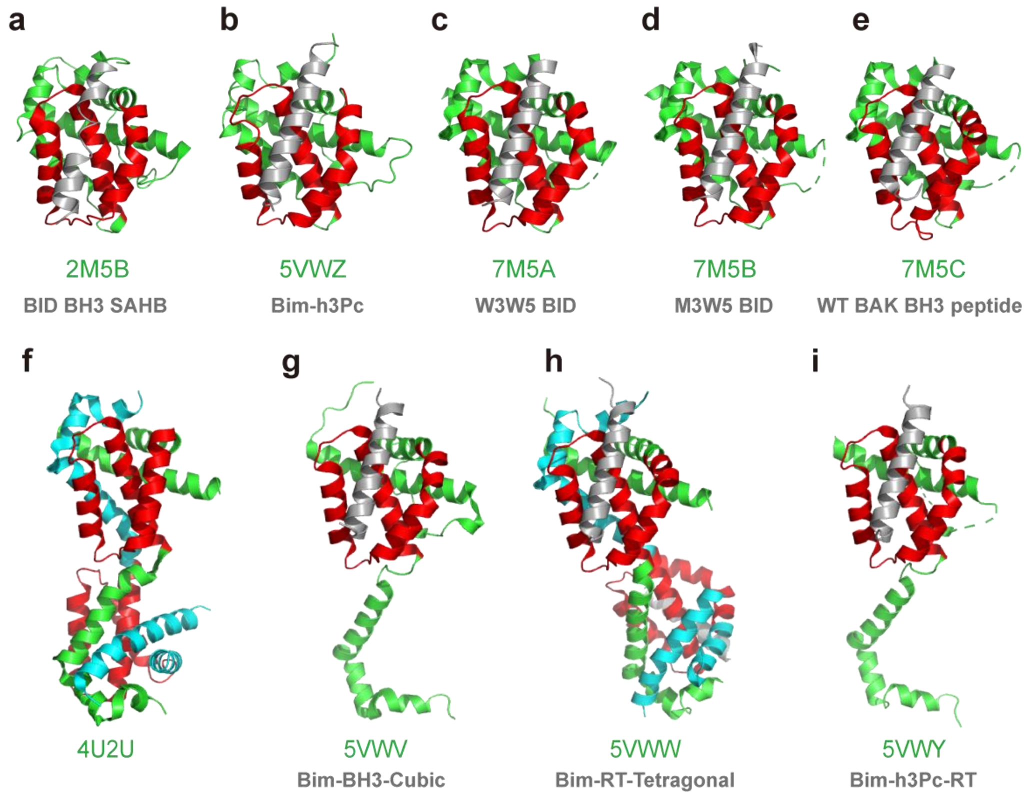

Supplementary Figure 4. The cartoon structures of BAK bound with different BH3 activator peptides. PDB codes are shown below the corresponding structures. The α3-α5 helices forming the hydrophobic groove of BAK are colored in red, and the bound peptides are colored in gray. The structures in green and cyan in (f-i) panels stand for the swapped domains of different BAK molecules.

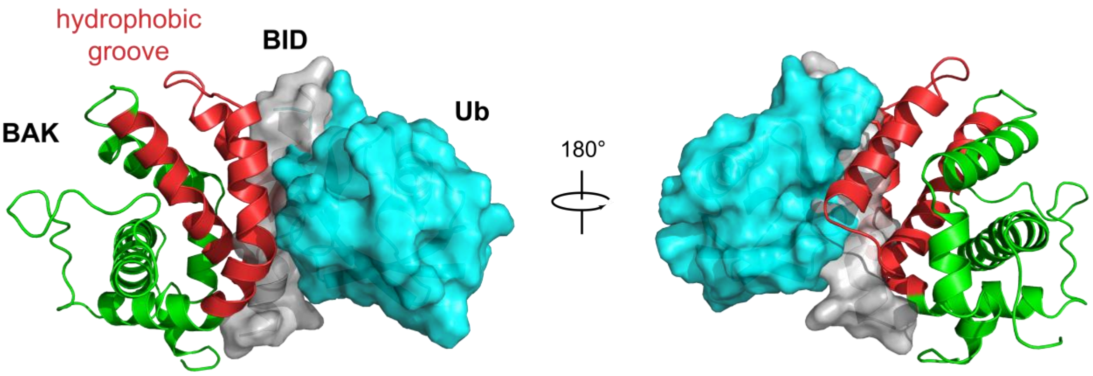

Supplementary Figure 5. The superimposed structures of Ub^{G76C}~BAK^{K113C} and BAK/BID complex. BAK in the previously reported BAK/BID complex structure (2M5B.PDB) is superimposed to the BAK^{K113C} subunit in Ub^{G76C}~BAK^{K113C} structure with an RMSD of 1.97 Å, and only BID (gray surface representation) is represented for clarity. The K113-conjugated Ub subunit is shown as cyan surface.

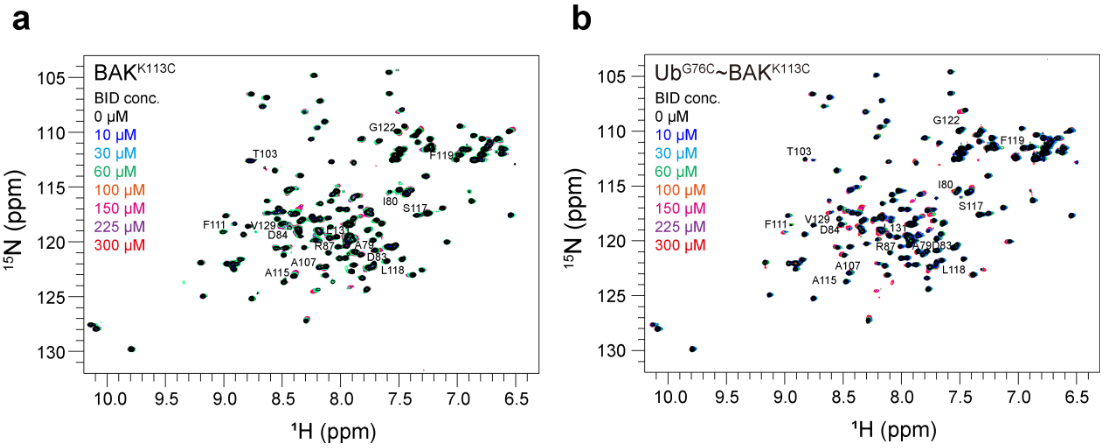

Supplementary Figure 6. (**a**) The superimposed ^{1}H-^{15}N HSQC spectra of 100 μM ^{15}N-labeled BAK^{K113C} and (**b**) 100 μM Ub^{G76C}~BAK^{K113C} (^{115}N-labeled BAK^{K113C} subunit and unlabeled Ub^{G76C}) monitoring titrations of BID BH3 peptide. The ^{1}H-^{15}N CSPs of 100 μM ^{15}N-labeled BAK^{K113C} or 100 μM Ub^{G76C}~BAK^{K113C} (with ^{15}N-labeled BAK^{K113C} subunit) upon addition of 300 μM BID BH3 peptide are plotted against BAK residue number in main Figure 2c and 2d, respectively. The hydrophobic groove residues, whose CSPs are used to determine the K_{D} values in main Figure 2e and 2f, are indicated in the spectra.

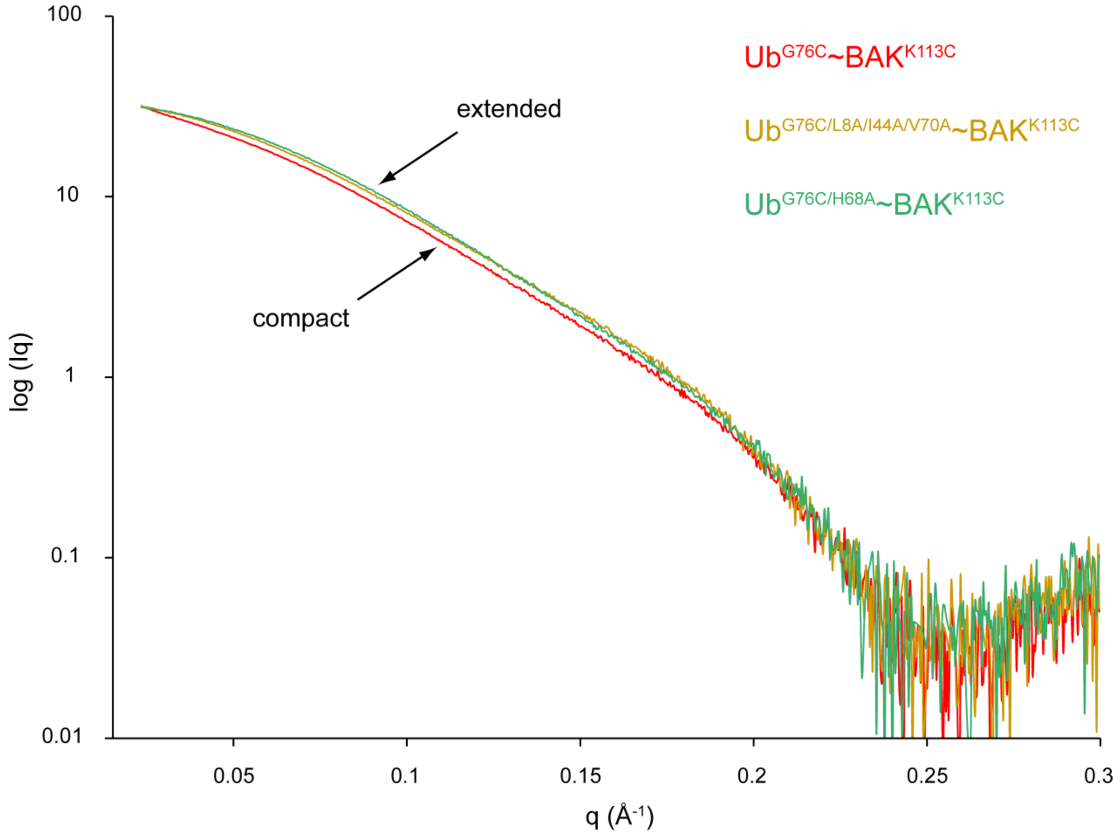

Supplementary Figure 7. Small-angle X-ray scattering (SAXS) analysis. The scattering data of Ub^{G76C}~BAK^{K113C}, Ub$^{G76C/L8A/I44A/V70A}$~BAK^{K113C} and Ub$^{G76C/H68A}$~BAK^{K113C} are represented in red, yellow and green, respectively. All data were scaled by the first point.

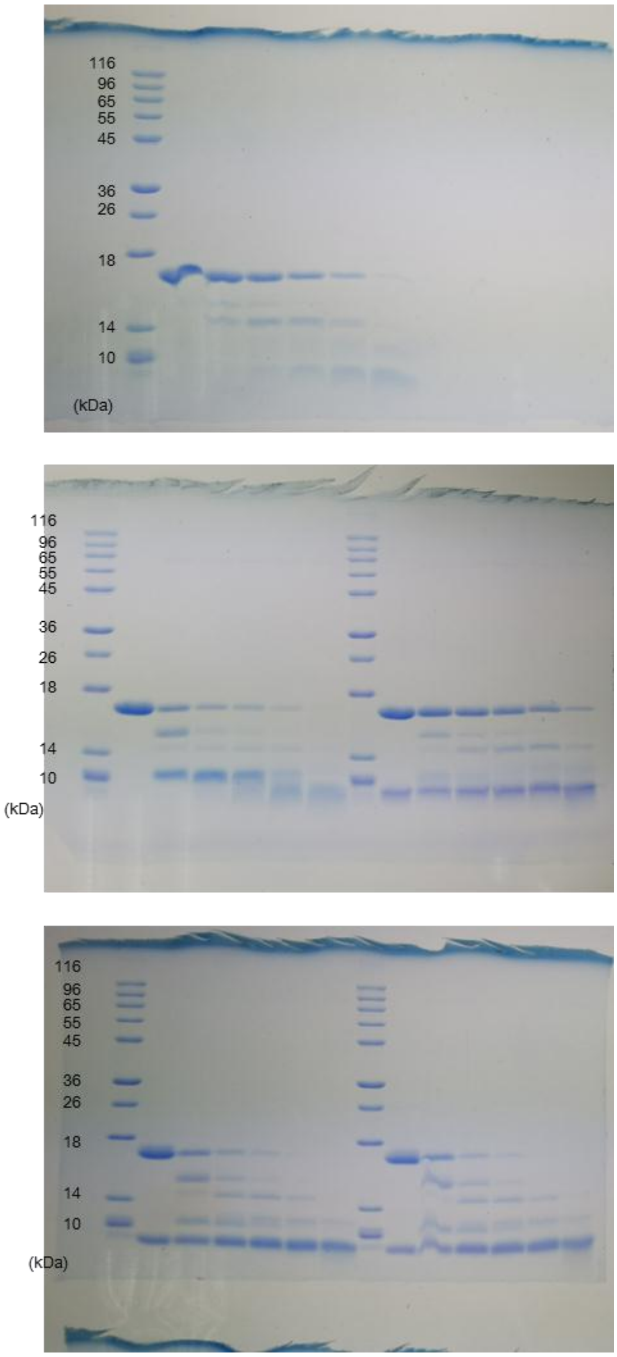

Supplementary Figure 8. The uncropped and unedited SDS-PAGE gels of Figure 4a in the main text.

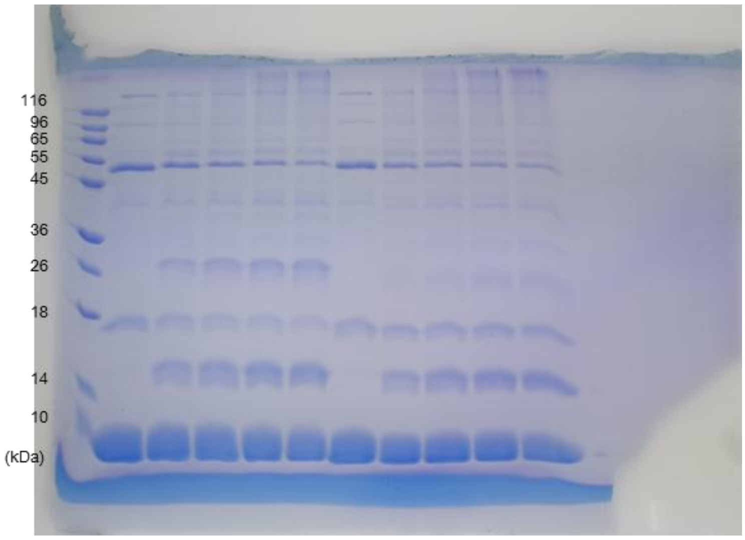

Supplementary Figure 9. The uncropped and unedited SDS-PAGE gel of Supplementary Figure 1.

Supplementary Table 1.

The NOE-derived distance restraints used for the structure calculation of Ub^{G76C}~BAK^{K113C}

Proton assignment in F_2 (BAK^{K113C})	Proton assignment in F_1 (Ub^{G76C})	Distance restraints (Å)
Y89 Hβ*	R42 Hγ*	3.8
Y89 Hβ*	V70 Hγ*	3.5
E92 Hβ*	V70 Hγ*	3.5
E92 Hγ1	V70 Hγ*	3.8
E92 Hγ2	V70 Hγ*	3.5
M96 Hε9	R42 Hγ*	3.2
M96 Hε9	R42 Hδ*	3.8
M96 Hε9	V70 Hγ*	3.2
I114 HδH1	R42 Hγ*	3.5
I114 HδH1	R42 Hδ*	4.2
I114 HδH1	V70 Hγ*	2.8
I114 Hγ2*	V70 Hγ*	2.8
L118 HδH1	R42 Hγ**	3.5
L118 HδH1	V70 Hγ*	2.8
