# Supplementary material for: Parkin-mediated ubiquitination inhibits BAK apoptotic activity by blocking its canonical hydrophobic groove
Source: Commun Biol. 2023 Dec 12;6:1260. doi: 10.1038/s42003-023-05650-z (PMC10716173; doi:10.1038/s42003-023-05650-z)
Supplement: Supplementary file 3 — Description of Additional Supplementary Files [file 42003_2023_5650_MOESM3_ESM.pdf]

Description of Additional Supplementary Files

File name: Supplementary Data 1
Description: Source data for Figures 4b and 4d.
